# CZT-based photon-counting-detector CT with deep-learning reconstruction: image quality and diagnostic confidence for lung tumor assessment

**DOI:** 10.1007/s11604-025-01759-9

**Published:** 2025-03-07

**Authors:** Tomoaki Sasaki, Hirofumi Kuno, Keiichi Nomura, Yoshihisa Muramatsu, Keiju Aokage, Joji Samejima, Tetsuro Taki, Eisuke Goto, Masashi Wakabayashi, Hideki Furuya, Hiroki Taguchi, Tatsushi Kobayashi

**Affiliations:** 1 Department of Diagnostic Radiology, National Cancer Center Hospital East, 6-5-1 Kashiwanoha, Kashiwa, Chiba 277-8577 Japan; 2 Department of Medical Information, National Cancer Center Hospital East, 6-5-1 Kashiwanoha, Kashiwa, Chiba 277-8577 Japan; 3 Department of Thoracic Surgery, National Cancer Center Hospital East, 6-5-1 Kashiwanoha, Kashiwa, Chiba 277-8577 Japan; 4 Department of Pathology and Clinical Laboratories, National Cancer Center Hospital East, 6-5-1 Kashiwanoha, Kashiwa, Chiba 277-8577 Japan; 5 Clinical Research Support Office, National Cancer Center Hospital East, 6-5-1 Kashiwanoha, Kashiwa, Chiba 277-8577 Japan; 6 Canon Medical Systems Corporation, 1385 Shimoishigami, Otawara, Tochigi 324-8550 Japan

**Keywords:** Cadmium–zinc–telluride, Computed tomography, Lung tumor, Photon-counting detector

## Abstract

**Purpose:**

This is a preliminary analysis of one of the secondary endpoints in the prospective study cohort. The aim of this study is to assess the image quality and diagnostic confidence for lung cancer of CT images generated by using cadmium–zinc–telluride (CZT)-based photon-counting-detector-CT (PCD-CT) and comparing these super-high-resolution (SHR) images with conventional normal-resolution (NR) CT images.

**Materials and methods:**

Twenty-five patients (median age 75 years, interquartile range 66–78 years, 18 men and 7 women) with 29 lung nodules overall (including two patients with 4 and 2 nodules, respectively) were enrolled to undergo PCD-CT. Three types of images were reconstructed: a 512 × 512 matrix with adaptive iterative dose reduction 3D (AIDR 3D) as the NR_AIDR3D_ image, a 1024 × 1024 matrix with AIDR 3D as the SHR_AIDR3D_ image, and a 1024 × 1024 matrix with deep-learning reconstruction (DLR) as the SHR_DLR_ image. For qualitative analysis, two radiologists evaluated the matched reconstructed series twice (NR_AIDR3D_ vs. SHR_AIDR3D_ and SHR_AIDR3D_ vs. SHR_DLR_) and scored the presence of imaging findings, such as spiculation, lobulation, appearance of ground-glass opacity or air bronchiologram, image quality, and diagnostic confidence, using a 5-point Likert scale. For quantitative analysis, contrast-to-noise ratios (CNRs) of the three images were compared.

**Results:**

In the qualitative analysis, compared to NR_AIDR3D_, SHR_AIDR3D_ yielded higher image quality and diagnostic confidence, except for image noise (all *P* < 0.01). In comparison with SHR_AIDR3D_, SHR_DLR_ yielded higher image quality and diagnostic confidence (all *P* < 0.01). In the quantitative analysis, CNRs in the modified NR_AIDR3D_ and SHR_DLR_ groups were higher than those in the SHR_AIDR3D_ group (*P* = 0.003, <0.001, respectively).

**Conclusion:**

In PCD-CT, SHR_DLR_ images provided the highest image quality and diagnostic confidence for lung tumor evaluation, followed by SHR_AIDR3D_ and NR_AIDR3D_ images. DLR demonstrated superior noise reduction compared to other reconstruction methods.

## Introduction

Photon-counting detector (PCD)-CT is an emerging technique that can drastically alter clinical practice. PCD-CT reportedly has several advantages over energy-integrated detector-CT (EID-CT), including improved spatial resolution, reduced image noise, multi-energy spectral imaging, and reduced radiation exposure [1–13]. The usefulness of PCD-CT for chest imaging has been increasingly reported, not only for the evaluation of normal structures [11–13] but also for interstitial pneumonitis [14, 15], pulmonary embolism [16, 17], and low-dose lung cancer screening [18]. However, to our knowledge, its use in lung tumor evaluation is rare.

Lung cancer presents various histological types, which require different treatment strategies [19, 20]. Lung adenocarcinoma, which is the most common form of lung cancer, is a heterogeneous tumor. The ratio of high-grade components in early lung adenocarcinomas must be assessed for prognostic analysis in pathological diagnosis [19, 20]. Although preoperative and pretreatment histologic assessment and lung cancer grading using CT is limited, several developments in preoperative imaging diagnosis have been made in recent years, mainly for early stage lung cancer [21–24]. Yanagawa et al. reported that high-resolution CT imaging evaluation of early stage lung adenocarcinoma is useful for estimating the invasive cancer component [21], which demonstrates the clinical utility of improved spatial resolution in lung cancer imaging.

Recently published phantom experiments on preclinical cadmium–zinc–telluride (CZT)-based PCD-CT prototypes have reported improvements in spatial resolution and iodine contrast, with a reduction in image noise [6]. However, there have been few reports on CZT-based PCD-CT evaluation in oncology. Moreover, the deep-learning reconstruction (DLR) algorithm is a significant image noise reduction technique with preserved spatial resolution, as compared to iterative reconstruction algorithms [25–29]. We hypothesized that super-high-resolution (SHR) CT images, such as those obtained with CZT-based PCD-CT, combined with the DLR technology, may have the potential to reduce image noise and improve spatial resolution in lung tumor evaluation as compared to normal-resolution (NR) CT images. We planned a prospective study to demonstrate that CZT-based PCD-CT would improve diagnostic performance of lung cancer evaluation as a primary endpoint. As the first step, this study aimed to assess the image quality and diagnostic confidence achieved with CT images generated using CZT-based PCD-CT for lung tumors, as a secondary analysis in a prospective study.

## Methods

Canon Medical Systems (Otawara, Japan) provided the PCD-CT system under a research agreement with our hospital. All data in our study were acquired and controlled by the authors, who did not receive financial support from Canon Medical Systems.

### Phantom study

A Catphan600 CTP528 phantom (The Phantom Laboratory, Salem, NY, USA) with slit widths ranging 16–21 lp/cm was scanned using a CZT-based PCD-CT system (TSX-501R, Canon Medical Systems), using the helical mode at 120 kVp, 250 mA (12.5 mGy), 0.5-s rotation, and a pitch factor of 0.6. The focal spot size was 0.4 mm × 0.5 mm. CT images were reconstructed as follows: For NR_AIDR3D_, SHR_AIDR3D_, and SHR_DLR_ images, 100 mm of field of view (FOV), a 512 × 512 matrix with 64 × 0.6-mm collimation and 1.8-mm slice thickness with adaptive iterative reconstruction 3D (AIDR 3D), 1024 × 1024 matrix using 192 × 0.2-mm collimation and 1.8-mm slice thickness with AIDR 3D, and 1024 × 1024 matrix with DLR were used, respectively.

### Study cohort

This study analyzed the secondary endpoint, image quality, of a prospective cohort study evaluating lung cancer diagnostic accuracy using CZT-based PCD-CT as an exploratory master protocol in oncology, which was approved by our institutional review board (jRCTs032220618). The primary endpoint analysis to evaluate the diagnostic specificity for lung cancer, as compared with a histopathological diagnosis, is planned for January 2026. The clinical usefulness of PCD-CT was determined by increased specificity, particularly in diagnostic accuracy [30, 31].

**Fig. 1:**
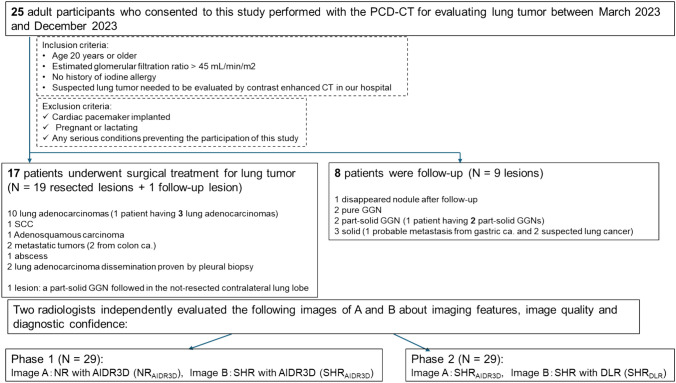
Schematic representation of this study

Study participants included patients suspected of having lung cancer requiring initial CT or pretreatment evaluation between February and December 2023. Written informed consent was obtained from all patients. Inclusion criteria were age ≥20 years, estimated glomerular filtration ratio ≥45 mL/min/m^2^, no iodine allergy history, and suspected lung cancer requiring contrast-enhanced CT evaluation at our hospital. Exclusion criteria included implanted cardiac pacemakers, pregnancy or lactation, and any serious condition preventing participation in the study (Fig. 1).

### CT protocol

After a plain chest CT scan, participants were injected with 600 mg I/kg iodine contrast medium (Omnipaque 300 injection syringe; GE Healthcare Pharma, Tokyo, Japan) at 2.5 mL/s into the antecubital vein. They underwent whole-chest scans, up to the pelvis, 80 s after injection initiation. The scan parameters were helical mode at 120 kVp, 250 mA (9.7 mGy), 0.5-s rotation, and pitch factor of 0.8. The focal spot size was 0.4 mm × 0.5 mm. CT images were reconstructed using NR_AIDR3D_ with a lung kernel (FC51) at 1.2-mm slice thickness and SHR_AIDR3D_ and SHR_DLR_ with lung kernels (FC54 and DLR lung, respectively) at 0.2-mm slice thickness (Table 1). The median FOV was 20 cm (ranging from 18 to 22 cm).

**Table 1:** Technical specification overview of our CZT-based PCD-CT system

	Technical specification overview
System	CZT-based photon-counting detector CT
Platform	Canon Aquilion Precision
Detector material	Cadmium–Zinc–Telluride
Scan field-of-view	Max. 500 mm
Scan mode	Axial, Helical
Tube voltage	120 kVp
Tube current	250 mA
Rotation time	Max. 0.35 s/rotation
Collimation	NR mode: 64 × 0.6 mm SHR: 192 × 0.2 mm (~40 mm @ isocenter)
Focal size	S2: 0.4 × 0.5 mm
Readout mode	NR mode: 3 × 3 SHR mode: 1 × 1

*CZT* cadmium–zinc–telluride, *CT* computed tomography, *NR* normal resolution, *SHR* super-high resolution

The DLR was initially developed as a prototype based on ultra-high resolution EID-CT data [32–35] and then optimized to the PCD-CT system. The DLR process comprises three main components: data domain filtering, hybrid iterative reconstruction (IR), and a deep convolutional neural networks (DCNN)-based restoration module [32, 34, 36]. For PCD-CT implementation, modifications were made to the pre-processing components (data domain filtering and hybrid-IR) of the DCNN architecture while maintaining the core restoration framework developed using ultra-high resolution EID-CT data.

### Qualitative image analysis

For each lung nodule, a key slice of NR_AIDR3D_ and SHR_AIDR3D_ (slice including the longest diameter of the solid portion) was determined by a chest radiologist (TS, with 20 years of experience). The key slice of SHR_DLR_ and SHR_AIDR3D_ was the same as only the post-processing reconstruction differed. The key slices of pairs of images A and B were compared side by side in random order on a custom-made program using commercially available software (MATLAB 2023b, MathWorks, Inc., Natick, MA, USA). Images A and B pairs were: (1) NR_AIDR3D_ and SHR_AIDR3D_ as phase 1, and (2) SHR_AIDR3D_ and SHR_DLR_ as phase 2. All images were displayed with window level −600 HU and window width 1600 HU on a commercially available display (RadiForce RX660, EIZO Corporation, Ishikawa, Japan). Because the image size and quality differed substantially among the image types, anonymization was impossible when two images were displayed side by side for both phases. Two chest radiologists (TS and HK with 17 years of experience) independently evaluated the first and second pairs (with at least 1-week interval) of image findings using the questions in Table 2 [21], and assessed image quality and diagnostic confidence using a 5-point Likert scale ranging from −2 to +2:

−2: Worse visibility in image B impacting on diagnostic interpretation
−1: worse visibility in image B without impacting diagnostic interpretation.
- 0: similar visibility in images A and B
- +1: better visibility in image B without impacting diagnostic interpretation
- +2: Better visibility on image B impacting diagnostic interpretation

**Table 2:** Questions regarding image findings

Number	Questions	Answer
Q1	Is the nodule pure ground-glass, part-solid, or solid nodule?	Pure GGO Part-solid nodule Solid nodule
Q2	How about the image noise?	NA****
Q3	Is the nodule lobulated?	Present or not
Q4	Is the nodule spiculated?	Present or not
Q5	Are the pleura entrapped?	Present or not
Q6	Is there vessel convergence?	Present or not
Q7	Is an air bronchiologram (AB) present?	Present or not
Q7-1*	Is the AB irregular?	Present or not
Q7-2*	Is the AB interrupted?	Present or not
Q7-3*	Is the AB dilated?	Present or not
Q8-1**	Is the ground-glass opacity (GGO) homogenous?	Present or not
Q8-2**	Is the GGO reticulated?	Present or not
Q8-3***	Is the solid part irregular?	Present or not
Q8-4***	Is the solid part centralized?	Present or not

*If Question 7 is marked “present,” answer this question

**If the nodule is a pure ground-glass nodule or is a part-solid nodule (Question 1), answer this question

***If the nodule is a part-solid nodule (Question 1), answer this question

****For Question 2, use a 5-point Likert score

The radiologists could view whole-CT images on a commercial DICOM viewer (RadiAnt DICOM Viewer version 2023.1, Medixant Inc., Poznan, Poland) to assess the whole tumor, if necessary.

### Quantitative image analysis

To evaluate the image noise effect, the contrast-to-noise ratios (CNRs) of the three images were compared. To assess spatial resolution improvement of the air bronchiolograms, the quantitative values related to the air bronchiolograms were compared. Because the partial volume effect is eliminated, the minimum CT value in the air bronchiologram could be close to the air density value, and boundary between the air bronchiologram and surrounding tumor could be more clearly delineated.

Because the NR_AIDR3D_ image had a 512 × 512 matrix, we expanded this image to a 1024 × 1024 matrix using bicubic interpolation for strict comparison and termed this the modified NR_AIDR3D_ (mNR_AIDR3D_) image (Supplemental Figure S1). Key slices of each nodule, previously defined in the qualitative analysis, were used for evaluation. One chest radiologist (TS) segmented the lung and tumor images using commercially available software (imageSegmenter on MATLAB R2023b, MathWorks, Inc.). The tumor was delineated using the graph cut function, and the lungs were segmented based on the threshold method. We define the CNR as follows:

CNR = (mean CT value of the tumor − mean CT value of the lung)/standard deviation of the CT value of the lung in the key slice.

When the tumor had an air bronchiologram according to phase 2 in the qualitative analysis, the radiologist drew a short line across the bronchioles on the SHR_AIDR3D_ image in the x- or y-axis directions to create a profile curve in the key slice (Supplemental Figure S1). The location of the line was copied to the key slices of the mNR_AIDR3D_ and SHR_DLR_ images. The minimum CT value was determined in the bronchioles, which should ideally have a CT value close to that of air. The difference between the adjacent CT values of the profile curve was defined as the tilt curve. The unit of the tilt curve was converted from pixels to millimeters to reduce the effects caused by differences in the field-of-view originating from body size. The minimum CT value, and maximum and minimum tilt values of the air bronchiologram among the three image types were compared.

### Statistical analyses

This is a preliminary analysis of one of the secondary endpoints of the main study, the primary endpoint of which was specificity. For the primary endpoint, the number of true-negative cases required to detect a 15% improvement to 75% of the previously reported dual-energy CT studies [30, 31] with a one-sided alpha of 0.1 and a power of 0.7, was 37. Because the true-negative cases were assumed to account for 70%, the planned sample size was set at 55.

For categorical image data, the kappa coefficients for each question were calculated to assess interobserver agreement between the two readers. The McNemar test was used to evaluate the percentage change in scores between the two assessments. Kappa coefficients were classified as indicating poor (k = 0.00–0.20), fair (k = 0.21–0.40), moderate (k = 0.41–0.60), good (k = 0.61–0.80), or excellent (k = 0.81–1.00) agreement [21]. For continuous data, such as the 5-point Likert scale, CNR, and tilt values of the air bronchiolograms, the Wilcoxon rank-sum test and Friedman test were performed. Statistical significance was set at *P* < 0.05. All statistical analyses were performed using Statistical Package for the Social Sciences version 25 for Windows (IBM Inc., Armonk, NY, USA).

## Results

### Phantom study

The slit separation in the SHR images was clearer than that in the NR_AIDR3D_ image, and the SHR_DLR_ image further improved slit separation compared to the SHR_AIDR3D_ image (Supplemental Figure S2).

### Study participants

This study included 25 participants (median age, 75 years, interquartile range 66–78 years, 18 men and 7 women) who underwent PCD-CT to evaluate lung tumors (Fig. 1). Subsequently, 17 patients (19 lesions) underwent surgical treatment, and the histopathological diagnosis was confirmed as follows: 10 patients with 12 lung adenocarcinomas (1 patient with 3 lung adenocarcinomas), 1 with squamous cell carcinoma, 1 with adenosquamous carcinoma, 1 with an abscess, 2 with metastatic tumors from colon cancer, and 2 lung adenocarcinoma disseminations confirmed by pleural biopsy. One patient who underwent resection in the right upper lobe had a persistent part-solid ground-glass nodule (GGN) in the contralateral lung lobe, which was confirmed on the 7-month follow-up CT and was also included in the analysis. Therefore, 20 lesions were investigated in this study.

Furthermore, eight patients with nine lesions were treated with follow-up CT at least 3 months later (median, 3 months). Follow-up CT images revealed that two patients persistently had a pure GGN, one had a part-solid GGN, one had two part-solid GGNs, and three had a solid nodule (one probable metastasis from gastric cancer and two probable lung cancers). However, in one patient, the nodule had disappeared during the follow-up period. Consequently, 29 lesions were identified for image analysis. The median whole tumor maximum size was 18.9 mm (interquartile range, 13.0–24.3 mm).

Among the 25 participants that underwent CZT-based PCD-CT, the mean volume-weighted CT dose index and dose-length product of the whole-CT scans were 19.1 ± 0.9 mGy and 1019.4 ± 127.0 mGy*cm, respectively.

### Qualitative image analysis

**Fig. 2:**
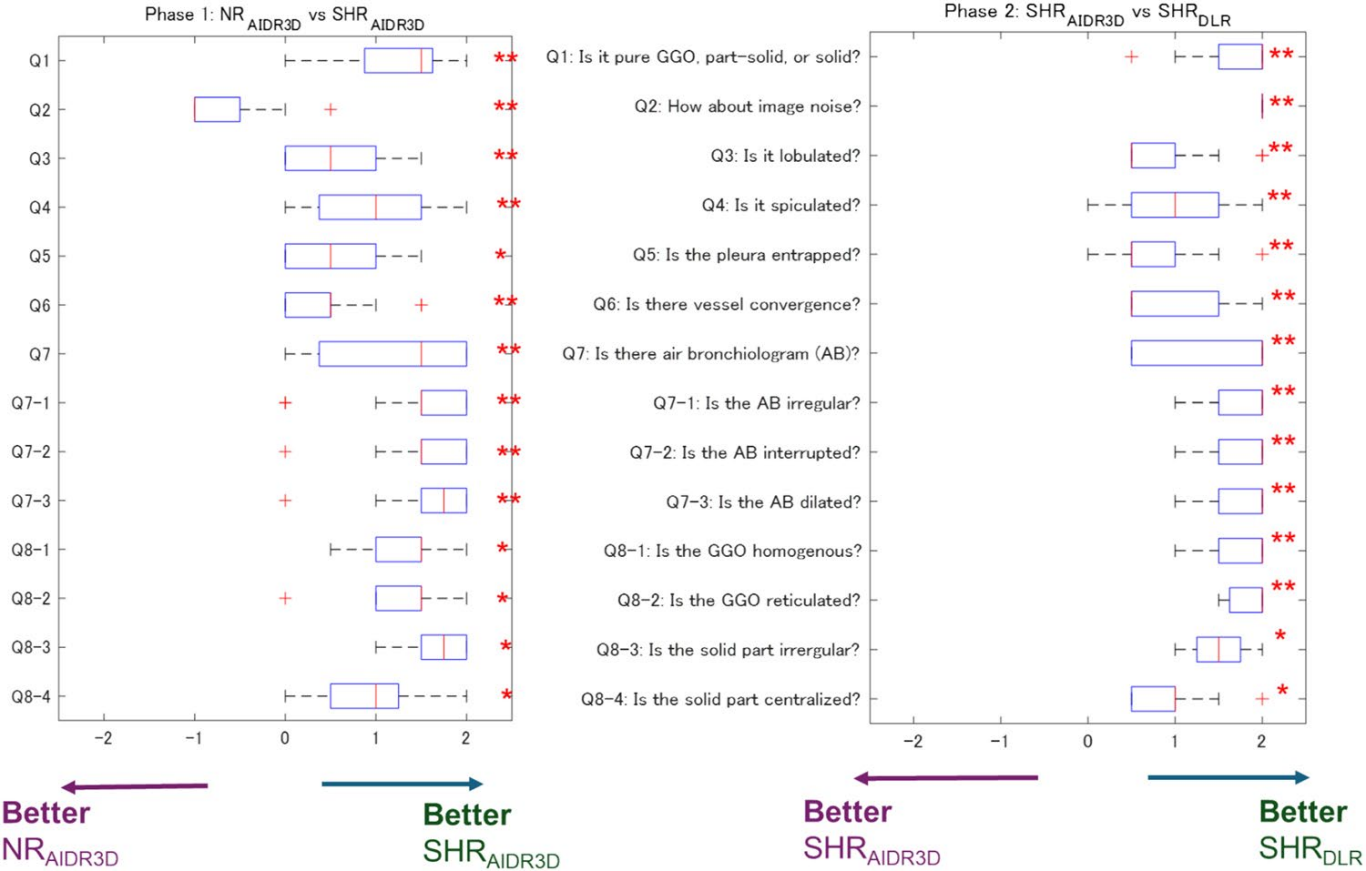
Box plots of reader-averaged scores for reader evaluation in **a** phase 1 and **b** phase 2. Reader-averaged scores are presented on the horizontal axis. The neutral score was zero. In phase 1, most median scores, except for Q2 (about image noise), were positive, which indicates that the image quality and diagnostic confidence of SHR_AIDR3D_ were better than those of NR_AIDR3D_. For image noise, the score was negative, which indicates that SHR_AIDR3D_ fared worse than NR_AIDR3D_. In phase 2, all median scores were positive, which means that SHR_DLR_ fared better than SHR_AIDR3D_. Symbols on the right side of each question indicate a significant difference by a two-sided one-sample Wilcoxon rank-sum test. *P < 0.01, **P < 0.001. NR_AIDR3D_, normal resolution with adaptive statistical iterative reconstruction 3D; SHR_AIDR3D_, super-high resolution with adaptive statistical iterative reconstruction 3D; SHR_DLR_, super-high resolution with deep learning reconstruction

For phase 1 (NR_AIDR3D_ vs. SHR_AIDR3D_), the kappa coefficients of the two radiologists were mostly above 0.6, except for the questions about the irregularity of air bronchiolograms and the ground-glass opacity (GGO) reticulation (Table 3). The kappa coefficient for the irregularity of the solid part in the part-solid nodule was not available because one reader answered “present” for all cases. Most scores were higher for SHR_AIDR3D_ than for NR_AIDR3D_, except for image noise (all *P* < 0.01, Fig. 2).

**Table 3:** Kappa coefficients of the two readers

Number	Kappa coefficients of phase 1	Ratios of the number of “present” to the total in phase 1^†^	Kappa coefficients of phase 2	Ratios of the number of “present” to the total in phase 2^†^
Q1	1.000	3/29 12/29 14/29	1.000	3/29 12/29 14/29
Q2	NA****		NA****	
Q3	0.664	4/29	0.760	4/29
Q4	0.854	10/29	0.928	11/29
Q5	0.839	8/29	0.848	9/29
Q6	0.713	3/29	0.751	7/29
Q7	1.000	18/29	1.000	19/29
Q7-1	0.444	8/18	0.872	13/19
Q7-2	1.000	14/18	1.000	16/19
Q7-3	1.000	15/18	0.771	16/19
Q8-1	0.727	8/15	0.842	4/15
Q8-2	0.587	4/15	0.842	10/15^††^
Q8-3	NA^†††^	11/12	NA^†††^	12/12
Q8-4	0.800	8/12	0.800	8/12

^†^The number of “present” means consistently marked as “present” by both readers

^††^The ratio in phase 2 was significantly higher than that in phase 1 (*P* = 0.031, McNemar test)

^†††^The Kappa coefficient was not calculated because at least one of the readers responded to all questions with “present.”

^****^The Kappa coefficients were not calculated; only a 5-point Likert score was evaluated for Q2

For phase 2 (SHR_AIDR3D_ vs. SHR_DLR_), all kappa coefficients, except those related to the irregularity of the solid part in the part-solid nodules, for the reason stated above, exceeded 0.6 (Table 3). All scores related to image quality and diagnostic confidence for SHR_DLR_ than for SHR_AIDR3D_ (*P* < 0.01; Fig. 2).

**Fig. 3:**
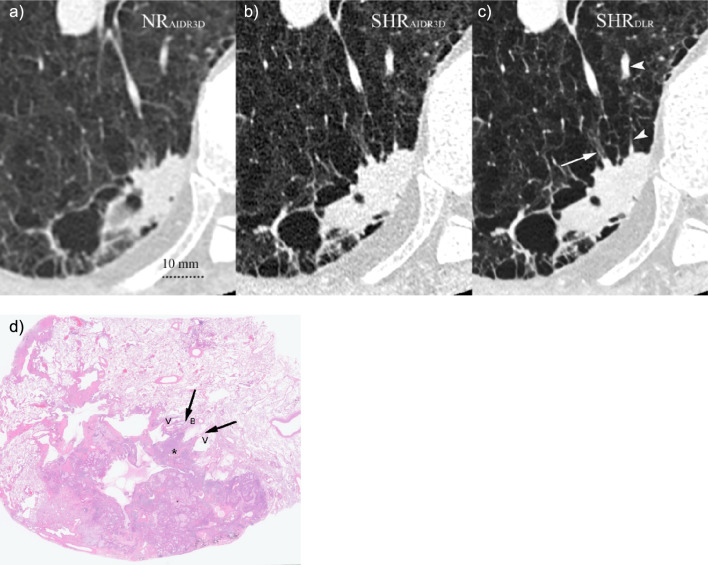
CT images of an 82-year-old man showing an irregular nodule in the right lower lung lobe: **a** NR_AIDR3D_, **b** SHR_AIDR3D_, and **c** SHR_DLR_ images. The nodule surrounded by emphysema appears irregular in **a** the NR_AIDR3D_ and is clearer in the **b** SHR_AIDR3D_. However, **c** the SHR_DLR_ image shows a dilated bronchiole with fluid collection entering the nodule (*arrow*) and another vessel (*arrowhead*) continuing to the proximal vein (*) coursing toward the center of the nodule, which is suggestive of vessel convergence. **d** Histopathological examination (overview) reveals an irregular tumor along the pleura diagnosed as invasive mucinous and non-mucinous adenocarcinoma with 50% papillary, 5% lepidic, 5% micropapillary, 30% acinar, and 10% complex glandular patterns. There is a locally spiculated part consisting of high-grade components (*) with vessel convergence (*arrows*). NR_AIDR3D_, normal resolution with adaptive statistical iterative reconstruction 3D; SHR_AIDR3D_, super-high resolution with adaptive statistical iterative reconstruction 3D; SHR_DLR_, super-high resolution with deep-learning reconstruction; V, vessel; B, bronchiole

**Fig. 4:**
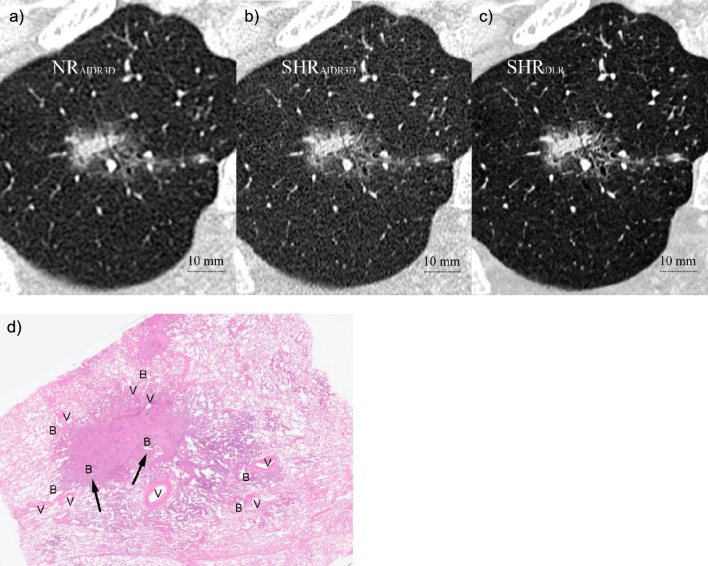
CT images of a 74-year-old man showing a part-solid nodule in the right upper lobe: **a** NR_AIDR3D_, **b** SHR_AIDR3D_, and **c** SHR_DLR_ images. Air bronchiolograms in the ground glass opacity are preserved among the three images. Irregularity of air bronchiolograms in the solid component is clearer in the **b** SHR_AIDR3D_ and **c** SHR_DLR_ images. Since the image noise is more prominent in the **b** SHR_AIDR_ image than in the **c** SHR_DLR_ image, the irregularity of the air bronchiologram appears clearer in the **c** SHR_DLR_ image. **d** Histopathological examination (overview) reveals that the tumor consists of 60% lepidic growth pattern (right part of the tumor) with preserved lung architecture and bronchiole and 40% invasive patterns (left part of the tumor; 20% papillary pattern, 15% acinar pattern, and 5% solid pattern) with fibrosis and obstruction of the bronchiole (*arrows*). NR_AIDR3D_, normal resolution with adaptive statistical iterative reconstruction 3D; SHR_AIDR3D_: super-high resolution with adaptive statistical iterative reconstruction 3D; SHR_DLR_: super-high resolution with deep-learning reconstruction; V: vessel; B: bronchiole

**Fig. 5:**
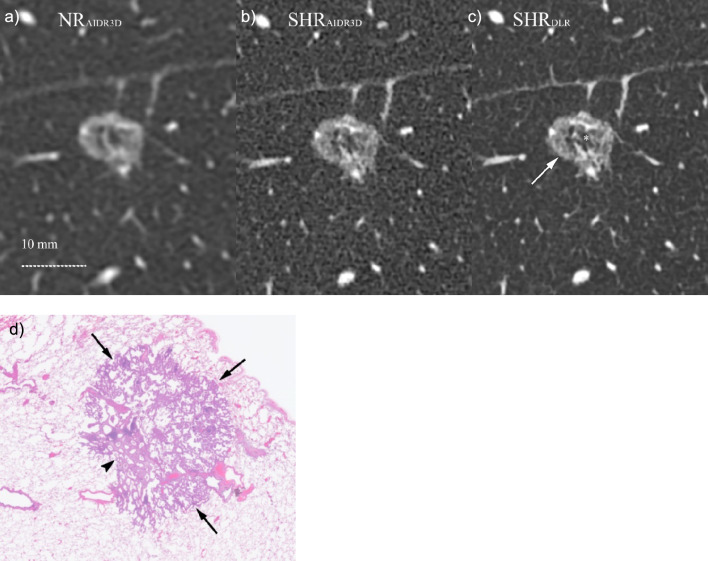
CT images of a 65-year-old woman showing a pure GGN in the right lower lobe: **a** NR_AIDR3D_, **b** SHR_AIDR3D_, and **c** SHR_DLR_ images. The ground glass opacity in the nodule (left upper side of the tumor) appears homogenous in **a** the NR_AIDR3D_ and equivocal in the **b** SHR_AIDR3D_ images. However, reticulation opacity (*arrow*) within the GGN is noted in **c** the SHR_DLR_ image. The air bronchiologram is also clearer (*) in the SHR_DLR_ image. **d** Histopathological examination (overview) reveals that most of the tumor consists of a lepidic growth pattern and appears to be a mixture of thickened alveolar septa and air space (*arrows*). Among them is an invasive component with an acinar pattern (*arrowhead*), resulting in the diagnosis of minimally invasive adenocarcinoma. NR_AIDR3D_, normal resolution with adaptive statistical iterative reconstruction 3D; SHR_AIDR3D_: super-high resolution with adaptive statistical iterative reconstruction 3D; SHR_DLR_: super-high resolution with deep-learning reconstruction; GGN, ground glass nodule

Overall, the lung nodule details were more frequently found in phase 2 than in phase 1, in particular, regarding vessel convergence (Fig. 3), irregularity of the air bronchiologram (Fig. 4), and GGO reticulation (Fig. 5; Table 3). Although the incidence of finding GGO homogeneity was decreased in phase 2, the incidence of GGO reticulation was increased in phase 2 compared to that in phase 1 (*P* = 0.031).

### Quantitative image analysis

The median CNRs were 2.69 in mNR_AIDR3D_, 2.58 in SHR_AIDR3D_, and 2.80 in SHR_DLR_ (Table 4). The CNRs of the three image types differed in the Friedman test (*P* < 0.001). In the post-hoc test, the CNRs of the mNR_AIDR3D_ and SHR_DLR_ images were higher than those of the SHR_AIDR3D_ images (*P* = 0.008 and *P* < 0.001, respectively).

**Table 4:** Comparison of the CNR and minimum CT value of air bronchiolograms

	mNR_AIDR3D_	SHR_AIDR3D_	SHR_DLR_
CNR	2.69 (1.72–4.30)*	2.58 (1.89–4.14)	2.80 (2.02–4.39)**
Minimum CT value of air bronchiologram (HU)	−710.7 (−591.4 to −906.2)	−982.5 (−839.5 to −1155)^†^	−969 (−857.75 to −1051.8)^††^

There were significant differences in the CNR and minimum CT values of the air bronchiologram among the three images (both *P* < 0.001, Friedman test)

*In the post-hoc test, the CNR in the mNRAIDR3D was significantly higher than that in SHRAIDR3D (*P* = 0.003)

**In the post-hoc test, the CNR in SHRDLR was significantly higher than that in SHRAIDR3D (*P* < .001)

^†^In the post-hoc test, the minimum CT value in SHR_AIDR3D_ was significantly lower than that in mNR_AIDR3D_ (*P* < .001)

^††^In the post-hoc test, the minimum CT value in SHR_DLR_ was significantly lower than that in mNR_AIDR3D_ (*P* = .004)

mNR_AIDR3D_: modified normal resolution with adaptive statistical iterative reconstruction 3D; SHR_AIDR3D_: super-high resolution with adaptive statistical iterative reconstruction 3D; SHR_DLR_: super-high resolution with deep-learning reconstruction

**Fig. 6:**
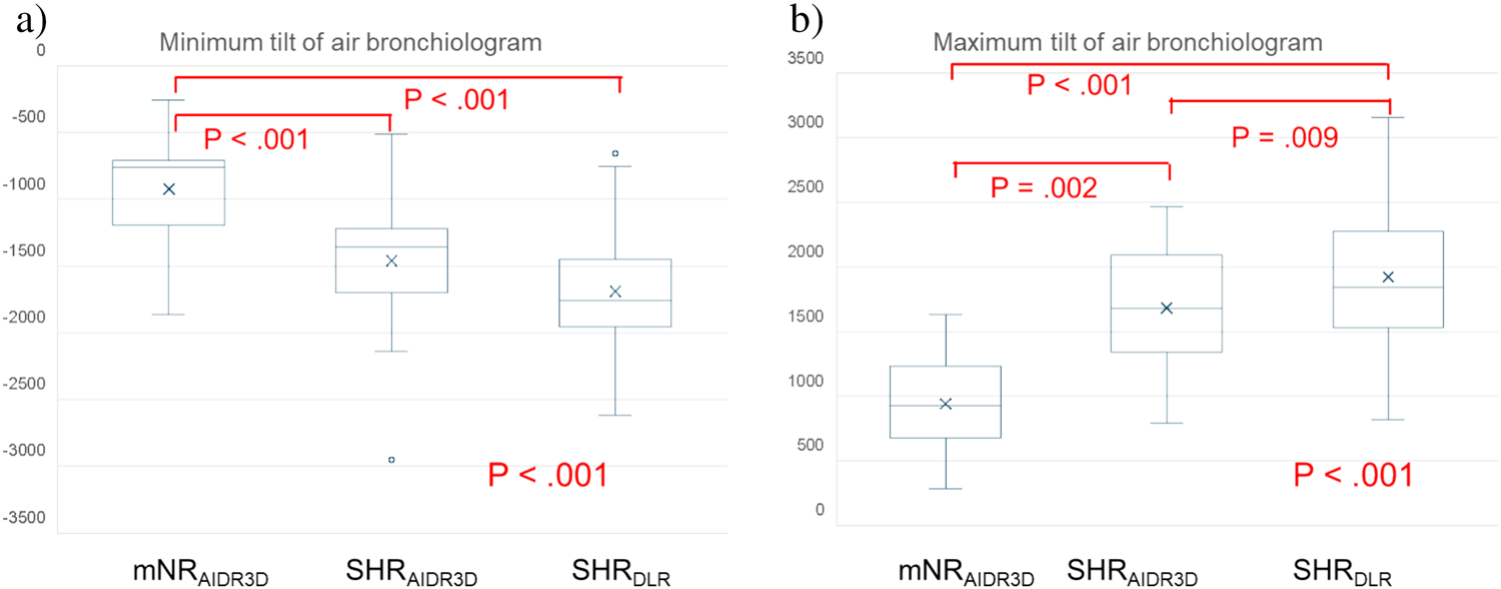
Box plots of the **a** minimum and **b** maximum tilt of air bronchiologram. **a** The three image groups differ significantly in both minimum and maximum tilts of the air bronchiologram by the Friedman test (both *P* < 0.001). **a** The minimum tilts of the SHR_AIDR3D_ and SHR_DLR_ images are significantly lower than those of mNR_AIDR3D_ images (both *P* < 0.001). **b** The maximum tilt of SHR_AIDR3D_ and SHR_DLR_ images are significantly higher than that of mNR_AIDR3D_ images (*P* = 0.002, and *P* < 0.001, respectively). Furthermore, that of SHR_DLR_ images are also significantly higher than that of SHR_AIDR3D_ images (*P* = 0.009). mNR_AIDR3D_, modified normal resolution with adaptive statistical iterative reconstruction 3D; SHR_AIDR3D_: super-high resolution with adaptive statistical iterative reconstruction 3D; SHR_DLR_: super-high resolution with deep-learning reconstruction

Nineteen lesions with air bronchiolograms were identified in phase 2. The minimum CT values differed among the three groups (*P* < 0.001, Table 3). In the post-hoc test, the minimum CT values of the air bronchiologram in the mNR_AIDR3D_ group were higher than those in the SHR_AIDR3D_ and SHR_DLR_ groups (*P* < 0.001 and *P* = 0.004, respectively). Moreover, the minimum and maximum tilts of the air bronchiologram were also differed among the three groups according to the Friedman test (*P* < 0.001 and *P* < 0.001, respectively). In particular, not only the tilts of the SHR_AIDR3D_ and SHR_DLR_ group were different from those of the mNR_AIDR3D_ group (all *P* < 0.01), but the maximum tilt of the SHR_DLR_ group was also higher than that of the SHR_AIDR3D_ group (*P* = 0.009; Fig. 6).

## Discussion

In the phantom experiment with photon-counting-detector-computed tomography, the spatial resolution was improved in the super-high-resolution images compared to that in the normal-resolution images. Additionally, the spatial resolution was further improved by using deep-learning reconstruction with super-high-resolution images. Quantitative image analysis revealed that super-high-resolution images, particularly those acquired with deep-learning reconstruction, successfully eliminated the partial volume effect of the air bronchiologram and reduced image noise. For qualitative image analysis, image quality and diagnostic confidence, but not image noise, were improved in the super-high-resolution images as compared to the normal-resolution images. The use of deep-learning reconstruction with super-high-resolution images not only reduced image noise but also further improved image quality, diagnostic confidence, and kappa coefficients in some image findings, and allowed discernment of reticulation in what had appeared to be homogeneous ground-glass opacities. Super-high-resolution images with deep-learning reconstruction obtained via photon-counting-detector-computed tomography can provide high spatial resolution images with remarkably improved image quality, resulting in increased diagnostic confidence.

Both image quality and diagnostic confidence have been considerably improved with PCD-CT compared to those of EID-CT [11, 37, 38]. This may be because PCD-CT is able to generate CT images using smaller collimation sizes, which improves the spatial resolution. Using a small focal spot in the X-ray tube current system can also contribute to improved spatial resolution [21, 39]. A narrow photon beam generated with a smaller focal spot size may be sufficiently sharp to suppress X-ray blurring [39], which reduces the chance of charge sharing on the photon-counting detector [2]. Yanagawa et al. reported that a high-resolution CT with the same focal spot size of 0.4 mm × 0.5 and a 160 × 0.25-mm collimation size was capable of ultra-high-resolution imaging that could successfully capture changes in the internal structure of lung adenocarcinomas [21]. In this study, PCD-CT used both a small collimation size and a small focal spot size, which may underlie the high-spatial-resolution of the images, which were comparable to pathology images.

Even with a PCD-CT system, image noise increases when CT images are reconstructed into thinner slices [7]. In the SHR images, compared to the NR images, both a smaller pixel size and thinner slice thickness were used, which increased the image noise. Therefore, using DLR with SHR images may improve the quality of SHR images. Recently, the DLR algorithm has been developed as a remarkable image noise reduction technique with preserved spatial resolution, as compared to iterative reconstruction algorithms [25–28]. Interestingly, thinner slice images obtained with DLR may have superior in image quality than that of thicker slice images obtained with iterative reconstruction [28]. Additionally, DLR is advantageous in high-contrast tissues, such as the lungs, because of the expected improvement in spatial resolution [26]. Furthermore, our phantom study also supported the concept of improved spatial resolution after using DLR, which shows the potential of the previously developed DLR algorithm for application to PCD-CT.

Implementation of 1024 × 1024 matrix images in clinical practice necessitates careful consideration of several practical workflow aspects. These high-resolution images require approximately four times more storage capacity compared to conventional 512 × 512 matrix images. While we used 0.2-mm slice thickness in this experimental study to explore the maximum potential of high-resolution imaging, a slice thickness of approximately 1 mm might be more practical for routine clinical implementation to reduce the total data amount. Additionally, the viewing workstations must be capable of efficiently handling these large datasets, and many currently available clinical analysis software packages may not be fully compatible with 1024 × 1024 matrix images. To optimize the clinical workflow, we suggest a hybrid approach where both conventional and high-resolution images are available, with the 1024 × 1024 matrix images being particularly valuable in evaluating detailed analysis of part-solid nodules.

This study had some limitations. First, our clinical research using CZT-based PCD-CT in lung cancer cases has recently begun, and the present study analyzed one of the secondary endpoints. No optimal data for generating the DLR algorithm for the PCD-CT system were available, and the DLR algorithm had to be transferred from a previously developed system. In future, a new DLR algorithm suitable for PCD-CT could be developed after the accumulation of CT images, and further improvement in image quality and subsequent reduction of radiation doses might be achieved. Second, we did not obtain histopathological confirmation for any of the cases. Third, the radiation dose in PCD-CT may not have been as low as expected in this study. Because PCD-CT is still under development, PCD-CT scans were performed at a constant tube current without using an automatic exposure control. Once PCD-CT is equipped with an automatic exposure control, the radiation dose used should be statistically compared between PCD-CT and EID-CT. Fourth, the present study focused solely on the secondary endpoint—image quality assessment—and did not directly assess whether PCD-CT was useful for lung cancer diagnosis. This should be evaluated in conjunction with diagnostic specificity assessment of the primary endpoint. Fifth, we did not directly compare NR_AIDR3D_ image and SHR_DLR_ image. However, the step-wise comparisons (NR_AIDR3D_ vs. SHR_AIDR3D_, and SHR_AIDR3D_ vs. SHR_DLR_) in our study allowed us to better understand the individual contribution of each factor to image quality improvement. Sixth, our sample size in this study was relatively small. In the future, our findings should be validated in larger cohort studies.

In conclusion, compared to normal-resolution images, super-high-resolution images generated by cadmium–zinc–telluride-based photon-counting-detector-computed tomography can improve image quality and diagnostic confidence for lung tumor evaluation. Moreover, applying deep-learning reconstruction to super-high-resolution images can further improve image quality and diagnostic confidence by reducing image noise and may have the potential to improve the visualization of small structures within lung tumors.

## Abbreviations

AIDR 3D: Adaptive iterative dose reduction 3D
CNR: Contrast-to-noise ratio
CZT: Cadmium–zinc–telluride
DLR: Deep-learning reconstruction
EID: Energy-integrated detector
mNR: Modified normal resolution
NR: Normal resolution
PCD: Photon-counting-detector
SHR: Super-high-resolution
GGO: Ground-glass opacity
